# Does aortic valve repair in valve-sparing aortic root reconstruction compromise the longevity of the procedure?

**DOI:** 10.6061/clinics/2017(04)03

**Published:** 2017-04

**Authors:** Ricardo Ribeiro Dias, José Augusto Duncan, Fabrício José de Souza Dinato, Lucas Lacerda Araújo, Hugo Monteiro Neder Issa, Fábio Fernandes, Charles Mady, Fábio Biscegli Jatene

**Affiliations:** Cirurgia Cardiovascular, Instituto do Coracao (InCor), Hospital das Clinicas HCFMUSP, Faculdade de Medicina, Universidade de Sao Paulo, Sao Paulo, SP, BR

**Keywords:** Aortic Diseases, Aorta Thoracic, Cardiac Surgical Procedures, Aortic Aneurysm, Thoracic, Aortic Valve

## Abstract

**OBJECTIVES:**

The effect of performing aortic valve repair in combination with valve-sparing operation on the length of time for which patients are free from reoperation is unclear. The objective of this study was to determine if the performance of aortic valve repair during valve-sparing operation modified the freedom from reoperation time.

**METHODS:**

From January 2003 to July 2014, 78 patients with a mean age of 49±15 years underwent valve-sparing operation. Sixty-eight percent of these patients were male. Twenty-two (28%) aortic valve repair procedures were performed in this patient population. In the aortic valve repair + valve-sparing operation group, 77.3% of patients had moderate/severe aortic insufficiency, while in the valve-sparing operation group, 58.6% of patients had moderate/severe aortic insufficiency (ns = not significant). Additionally, 13.6% of patients in the aortic valve repair + valve-sparing operation group had functional class III/IV, while 14.2% of patients in the valve-sparing operation group had functional class III/IV (ns).

**RESULTS:**

The in-hospital and late mortality rates, for the aortic valve repair + valve-sparing operation and valve-sparing operation groups were similar, as they were 4.5% and 3.6%; and 0% and 1.8%, respectively. In the aortic valve repair + valve-sparing operation group, 0% of patients presented moderate/severe aortic insufficiency during late follow-up, while in the valve-sparing operation group, 14.2% of patients presented with moderate/severe aortic insufficiency during this period (ns). In the aortic valve repair + valve-sparing operation group, 5.3% of patients presented with functional class III/IV, while in the valve-sparing operation group, 4.2% of patients presented with functional class III/IV (ns). In the aortic valve repair + valve-sparing operation group, 0% of patients required reoperation, while in the valve-sparing operation group, 3.6% of patients required reoperation over a mean follow-up period of 1621±1156 days (75 patients).

**CONCLUSION:**

Valve-sparing operation is a safe and long-lasting procedure and performance of aortic valve repair when necessary does not increase risk of reoperation on the aortic valve.

## INTRODUCTION

The worldwide incidence and prevalence of thoracic aortic disease (TAD) has increased significantly during the last two decades, a finding that constrasts with those pertaining to abdominal aortic disease 1.

In São Paulo State, Dias et al. performed an epidemiological study on TAD, during which they observed a significant reduction in TAD-related mortality. However, these authors also found that there is a lack of information regarding the incidence and prevalence of TAD disease, whose mortality rate is high. Moreover, they noticed that patients with TAD are extremely difficult to manage, since local centers are often unable to effectively treat these patients, the referral of affected patients to centers specializing in TAD treatment is inefficient and the results of treatment provided by the centers to which patients are referred are often suboptimal 2.

The majority of patients with TAD suffer from diseases affecting the aortic root/ascending aorta. Aortic root reconstruction with a composite mechanical aortic valve graft is the operation most frequently performed for diseases of this segment of the aorta; however, much has been written about the aortic root replacement through valve-sparing operation (VSO) and the major difficulties related to the technical complexity and reproducibility of the procedure, as well as the benefits of preserving the native valve and the advantages of non-anticoagulation afforded to patients treated with VSO 3.

It is known that, when aortic root dilation occurs, aortic valve insufficiency secondary to annulus dilatation is usually observed. However, dysfunction of one or more leaflets of the aortic valve is also often present, with prolapse of the cusps into the left ventricle resulting in aortic regurgitation. This phenomenon is a frequent cause of valve failure and should be addressed when the aortic root is reconstructed during VSO. Thus, many have questioned whether performance of valve repair during VSO can compromise the longevity of the procedure free from reoperation 4.

The results of an echocardiographic analysis of the predictors of residual aortic regurgitation or recurrence of aortic insufficiency after VSO demonstrated that eccentric jets of aortic regurgitation, effective heights of the low free edges of valve cusps in relation to ventricular-aortic junction (VAJ) and contact between the low free edges of opposing leaflets below the VAJ are the predictors of immediate aortic insufficiency or during the early follow-up period and therefore should be properly evaluated during surgery 5.

Since VSO is considered the procedure of choice for treating aortic root disease 3,6,7, we recognize the necessity of demonstrating that performance of aortic valve repair (AVR) during VSO does not compromise the longevity of the procedure and does not affect the freedom from reoperation rate during follow-up. Therefore, the aim of this study was to evaluate whether performance of AVR during VSO changed the length of time for which patients are free from aortic valve reoperation.

## METHODS

From January 2003 to July 2014, 78 patients with or without aortic valve insufficiency underwent aortic root reconstruction using the VSO technique. Fifty-five patients underwent VSO using the reimplantation technique (70.5%), and 23 underwent VSO using the remodeling technique. Both groups were assessed together to evaluate the longevity of the procedure and were compared regarding their levels of aortic valve failure and their need for AVR (rather than being assessed as separate remodeling technique and reimplantation technique groups).

The patients were divided into two groups for analysis. Group 1 consisted of patients who underwent VSO without AVR (56 patients - 71.2%) and group 2 consisted of patients who underwent AVR + VSO (22 patients - 28.2%). Nineteen patients of group 2 underwent aortic root reconstruction using the reimplantation technique (86.4%).

Regarding aortic valve insufficiency etiologies, most cases of aortic valve regurgitation had developed secondary to annulus dilatation, aortic root enlargement or leaflet prolapse.

The demographic characteristics of the sample are comparatively listed in Table 1.

The choice to use one technique or the other for aortic root reconstruction was based on time more than on clinical motivation. Currently, the reimplantation technique is preferred due to the possibility that valve components can be fixed to a polyester tube, regardless of the presence of a genetic syndrome or acquired disease.

The cardiopulmonary bypass and myocardial ischemia times, as well as the frequencies with which associated procedures were performed, were similar between the two groups (Table 2).

Patients were followed-up prospectively and evaluated annually via transthoracic echocardiography studies. The mean follow-up time was 1621±1156 days and clinical and echocardiographic follow-up assessments were performed for 98.7% and 86.7% of patients in the AVR + VSO and VSO groups, respectively.

The study was approved by the Scientific and Ethics Committee of our institution and the requirement of written informed consent was waived, based on the characteristics of the study.

### Statistical analysis

The results were expressed as the mean±SD and as percentages.

In the analysis, the normality assumption was verified by the Kolmogorov-Smirnov test and visual data analysis.

When paired, continuous data were compared using Student's t test, and when not paired, continuous data were compared using the Mann-Whitney U test. Categorical data were analyzed using a chi-square test or Fisher's exact test.

Kaplan-Meier curves and the log-rank test were used to compare the survival rates of the VSO and AVR + VSO groups.

Propensity score: to reduce the bias resulting from the non-random collection of data at different times, balance the characteristics of the sample and comparatively analyze the patients who underwent reimplantation operations with and without AVR, we combined the 19 patients from the VSO and AVR + VSO groups into one group and evaluated the patients who underwent reimplantation technique using a propensity score, which was defined as the conditional probability of being treated. The most relevant confounding factors (age, left ventricular ejection fraction, degree of aortic insufficiency and classification of heart failure according to the New York Heart Association) were entered as predictors, and the corresponding tolerance margin was 0.05 of logit (the degree of aortic insufficiency and severity of heart failure according to the New York Heart Association classification could not be used since there was no significant association between these parameters and the probability of being treated, which prevented the use of the logistic regression model).

Differences were considered significant for *p*<0.05. All analyses were performed using IBM software - SPSS (version 21, IBM Corp. Armonk, NY, USA).

## RESULTS

With the exception of age, as we observed that patients undergoing AVR + VSO were older than patients undergoing VSO, the two groups were similar with respect to their clinical characteristics, such as their etiopathogenesis of aortic disease, their degrees of aortic insufficiency, their left ventricular ejection functions, their aortic sizes, their medical histories, their operative times and their associated procedures's complexities (Tables 1 and 2).

### In-hospital mortality and immediate postoperative complications

In-hospital mortality occurred in three patients (3.8%), including two in the VSO group and one in the AVR + VSO group (ns).

The main in-hospital complications were infection, which occurred in 22 patients (28%), including 9 patients who developed pneumonia, 5 patients who developed sepsis, 4 patients who developed superficial wound infections, 2 patients who developed tracheobronchitis and 2 patients who developed urinary tract infection; arrhythmias, particularly atrial fibrillation, which occurred in 11 patients (14%); and reoperations, which occurred in 9 patients (11.5%), including 6 patients who presented bleeding, one patient who developed cardiac tamponade and 2 patients who required compression bandages removal (Table 3). There were no significant differences in the incidences of these complications between the two groups.

### Late mortality

Two deaths occurred (2.6%) during the late follow-up period (both patients underwent VSO using the remodeling technique, without AVR). The first death, occurred secondary to acute myocardial infarction three years after surgery, and the second death occurred secondary to infective endocarditis eight years and four months after the procedure, (the patient was referred from another institution and was deemed unsuitable for surgery due to his poor clinical condition when he presented to our facility) (Table 4).

### Echocardiographic evaluation and functional class

Despite the significant difference between the two groups with respect to the time at which the patients underwent their echocardiographic evaluations, 100% of patients who underwent AVR + VSO presented with mild aortic valve regurgitation or less, while 77.8% of patients who underwent VSO presented mild aortic regurgitation or less (ns). In addition, 73.7% of patients in the AVR + VSO group presented with heart failure class II or less, while 52.1% of patients in the VSO presented with heart failure functional class II or less (ns) (Table 4).

### Survival and freedom from reoperation for aortic valve failure

**Figure 1:**
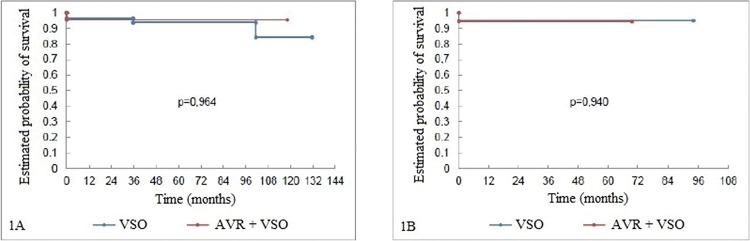
Fig 1A: Survival curves of the patients (Kaplan-Meier) undergoing aortic root reconstruction through AVR and non-AVR techniques. Fig 1B: Survival curves of the patients (Kaplan-Meier) undergoing aortic root reconstruction. The patients were paired according to their propensity scores.AVR=aortic valve repair; VSO=valve-sparing operation.

Despite the significant difference between the two groups with respect to the follow-up times during which patients underwent AVR + VSO and VSO were evaluated, there was no difference in late survival between the groups, regardless of the surgical technique utilized (Figure 1A and 1B).

**Figure 2:**
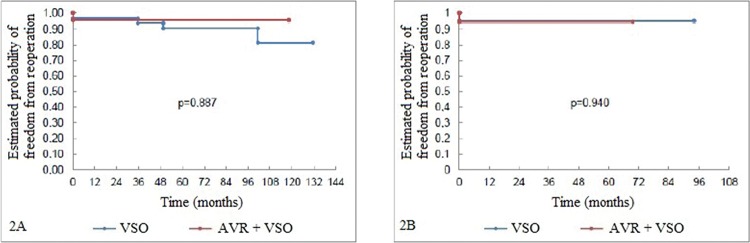
Fig 2A: Freedom from reoperation curve (Kaplan-Meier) pertaining to the aortic valves of the patients undergoing aortic root reconstruction through AVR and non-AVR techniques. Fig 2B: Freedom from reoperation curve (Kaplan-Meier) pertaining to the aortic valves of the patients undergoing aortic root reconstruction. The patients were paired according to their propensity scores.AVR=aortic valve repair; VSO=valve-sparing operation.

The freedom from reoperation rates were also similar between the groups (despite the difference in follow-up time between them). Only one patient underwent reoperation for aortic valve replacement. This procedure was performed four years after VSO without AVR operation (which was performed with the reimplantation technique) and the postoperative course was uneventful (Figure 2A and 2B).

## DISCUSSION

Whenever possible, VSO must be considered and performed, regardless of patient age and aortic disease etiopathogenesis, as the literature has consistently reported that patients who undergo VSO experience fewer complications related to anticoagulation or reoperations because of infection or prosthesis dysfunction than patients who undergo the Bentall procedure 6.

VSO requires knowledge of the complex anatomy of the functional aortic root and the relationship between its valve leaflets and the aortic wall and adjacent tissues, a portion of which are fibrous (fibrous skeleton of the heart) and another portion of which are muscular (interventricular septum). It is also crucial to determine the size of the aortic annulus, the location of the VAJ, the effective and geometric heights of the valve leaflets, the ideal levels and areas of cusp coaptation, and the height at which coaptation occurs relative to the location of the VAJ, as well as to understand the importance of the sinotubular junction and its height relative to that of the aortic annulus.

The main mechanisms underlying the development of aortic valve insufficiency are related to primary changes in the valve leaflets or to secondary changes imposed by aortic diseases, as stated in the classification proposed by El Khoury et al., which proposes the performance of specific repair procedures for each of these situations 8.

Currently, valve-sparing aortic root reconstruction operations are considered the procedures of choice for treating aortic root diseases because they enable native valve preservation. When necessary, however, additional procedures involving the aortic valve cusps may be performed if the leaflets are responsible for causing aortic regurgitation 3,7,9.

Albes, Stock and Hartrumpf reported a meta-analysis in which they found that performing VSO using the reimplantation technique, as proposed by David and Feindel 10, when aortic root reconstruction is necessary is associated with greater longevity, particularly if performed in patients with congenital abnormalities of the aortic wall, while performing VSO using the remodeling technique, as proposed by Yacoub et al. 11, seems to preserve the physiology of the spared valve more faithfully than the reimplantation procedure 12.

However, whether performing AVR along with VSO is a risk factor with respect to the longevity of the valve-sparing procedure remains controversial 13,14.

Thus, we developed this study, as our previous experience with VSO demonstrated that we could safely extend the operation for patients with primary valvular insufficiency (with or without aortic root dilation). For this reason, this subgroup of patients had a shorter follow-up time than the subgroup of patients who underwent VSO only.

The mortality rate was low in both groups, which did not differ with respect to other outcome parameters, findings similar to those reported in the literature. Additionally, it should be emphasized that 77% of patients who underwent valve repair presented moderate or severe aortic insufficiency on preoperative echocardiography (50% of patients had severe disease) and that no major insufficiencies were noted on the postoperative studies of these patients (i.e., zero patients had worse than mild valve regurgitation).

The limitations of this study were mainly related to the small sample of patients who underwent VSO at our facility, the small number of patients who underwent valve repair procedures and the short follow-up time during which this subgroup of patients was assessed.

Despite the abovementioned limitations, we can conclude that VSO is a safe and long-lasting procedure and performance of AVR when necessary does not increase the risk of late reoperation on the aortic valve.

## AUTHOR CONTRIBUTIONS

Dias RR was responsible for the manuscript writing and study design. Duncan JA and Dinato FJ were responsible for manuscript writing and acquisition of data. Araujo LA and Issa HM were responsible for acquisition of the data. Fernandes F and Mady C were responsible for data analysis and critical revision of the manuscript. Jatene FB was responsible for critical revision of the manuscript.

## Figures and Tables

**Table 1:** Preoperative characteristics of the patients in the AVR and non-AVR groups. The patients were paired according to their propensity scores.

	Original Cohort			Paired Cohort		
	Repair			Repair		
Variables	No (n=56)	Yes (n=22)	*p*	No (n=56)	Yes (n=22)	*p*
Mean age, years (mean ± SD)	48+15	56+14	0.046	57+14	57+14	0.991
Male, n (%)	38 (67.9)	14 (63.6)	0.722	14 (73.7)	12 (63.2)	0.485
BMI, kg/m2 (mean ± SD)	24+5	26+4	0.283	26+4	26+4	0.696
LVEF, % (mean ± SD)	0.61+0.10	0.61+0.10	0.944	0.57+0.09	0.60+0.10	0.258
LVEDV, (mean ± SD)	201+108	232+64	0.463	207+56	234+67	0.245
Aortic diameter, mm (mean ± SD)	56+9	56+7	0.954	57+9	57+7	0.951
**Risk factor**						
Marfan syndrome, n (%)	11 (19.6)	1 (4.6)	0.162	1 (5.3)	1 (5.3)	1.000
Bicuspid aortic valve, n (%)	3 (5.4)	1 (4.6)	1.000	-	-	-
Tricuspid aortic valve, n (%)	52 (92.9)	21 (95.5)	1.000	18 (94.7)	19 (100.0)	1.000
Hypertension, n (%)	42 (75.0)	18 (81.8)	0.520	16 (84.2)	15 (79.0)	1.000
Diabetes mellitus, n (%)	5 (8.9)	4 (4.6)	0.670	3 (15.8)	1 (5.3)	0.604
Dyslipidemia, n (%)	9 (16.1)	5 (22.7)	0.522	4 (21.1)	5 (26.3)	1.000
Hypothyreoidism, n (%)	1 (1.8)	1 (4.6)	0.487	1 (5.3)	1 (5.3)	1.000
Acute RF, n (%)	2 (3.6)	0 (0.0)	1.000	2 (10.5)	0 (0.0)	0.487
Chronic RF, n (%)	2 (3.6)	2 (9.1)	0.315	1 (5.3)	2 (10.5)	1.000
Dialytic RF, n (%)	1 (1.8)	0 (0.0)	1.000	-	-	-
Smoking, n (%)	22 (39.3)	11 (50.0)	0.389	9 (47.4)	10 (52.6)	0.746
Family history, n (%)	6 (10.7)	1 (4.6)	0.666	0 (0.0)	1 (5.3)	1.000
COPD, n (%)	2 (3.6)	2 (9.1)	0.315	1 (5.3)	1 (5.3)	1.000
Cancer, n (%)	1 (1.8)	0 (0.0)	1.000	1 (5.3)	0 (0.0)	1.000
Dyspepsia, n (%)	9 (16.1)	3 (13.6)	1.000	1 (5.3)	2 (10.5)	1.000
Coronary insufficiency , n (%)	8 (14.3)	6 (27.3)	0.201	4 (21.1)	5 (26.3)	1.000
Prior MI, n (%)	1 (1.8)	0 (0.0)	1.000	-	-	-
Chest pain, n (%)	19 (33.9)	9 (40.9)	0.563	7 (36.8)	8 (42.1)	0.740
Prior atrial fibrillation, n (%)	0 (0.0)	1 (4.6)	0.282	0 (0.0)	1 (5.3)	1.000
**Cardiac insufficiency, n (%)**			0.507			1.000
FC I, n(%)	36 (64.3)	12 (54.6)		11 (57.9)	11 (57.9)	
FC II, n (%)	12 (21.4)	8 (36.4)		6 (31.6)	7 (36.8)	
FC III, n (%)	6 (10.7)	2 (9.1)		2 (10.5)	1 (5.3)	
FC IV, n (%)	2 (3.6)	0 (0.0)		0 (0.0)	0 (0.0)	
**Indication for surgery, n (%)**						
Aneurysm	53 (94.6)	21 (95.5)	1.000	19 (100)	18 (94.7)	1.000
Type A chronic dissection	4 (7.1)	1 (4.6)	1.000	0 (0.0)	1 (5.3)	1.000
**Aortic valve function, n (%)**			0.384			0.489
absent AI	5 (9.3)	0 (0.0)		0 (0.0)	0 (0.0)	
minimum AI	2 (3.7)	0 (0.0)		0 (0.0)	0 (0.0)	
discrete AI	15 (27.8)	5 (22.7)		4 (21.1)	3 (15.8)	
moderate AI	16 (29.6)	6 (27.3)		9 (47.4)	6 (31.6)	
severe AI	16 (29.6)	11 (50.0)		6 (31.6)	10 (52.6)	
**Urgent/emergency surgery, n (%)**	0 (0.0)	1 (4.6)	0.282	0 (0.0)	1 (5.3)	1.000

AVR=aortic valve repair; BMI=body mass index; COPD=chronic obstructive pulmonary disease; FC=functional class (New York Heart Association); LVEF=left ventricular ejection fraction; LVEDV=left ventricular end-diastolic volume; MI=myocardial infarction; RF=renal failure; SD=standard deviation.

**Table 2:** Intraoperative variables of the patients in the AVR and non-AVR groups. The patients were paired according to their propensity scores.

	Original Cohort			Paired Cohort		
	Repair			Repair		
Variables	No (n=56)	Yes (n=22)	*p*	No (n=19)	Yes (n=19)	*p*
**Time of CPB, min (mean±SD)**	155±35	159±28	0.585	163±32	165±24	0.768
**Time of myocardial ischemia, min (mean±SD)**	133±29	139±23	0.369	139±25	145±17	0.458
**Aortic Approach**			0.054			-
Reimplantation	36 (64.3)	19 (86.4)				
Remodeling	20 (35.7)	3 (13.6)				
**Associated Procedures, n (%)**						
CABG, n (%)	5 (8.9)	1 (4.6)	0.670	2 (10.5)	1 (5.3)	1.000
Mitral Replacement/Repair, n (%)	1 (1.8)	2 (9.1)	0.190	0 (0.0)	2 (10.5)	0.487

AVR=aortic valve repair; CABG=coronary artery bypass grafting; CPB=cardiopulmonary bypass; SD=standard deviation.

**Table 3:** In-hospital postoperative outcomes of the patients in the AVR and non-AVR groups. The patients were paired according to their propensity scores.

	Original Cohort			Paired Cohort		
	Repair			Repair		
Variables	No (n=56)	Yes (n=22)	*p*	No (n=19)	Yes (n=19)	*p*
**Reoperation, n (%)**						
Bleeding	4 (7.1)	2 (9.1)	1.000	4 (21.1	2 (10.5)	0.660
Tamponade	0 (0.0)	1 (4.6)	0.282	0 (0.0)	1 (5.3)	1.000
Compresses removal	2 (3.6)	0 (0.0)	1.000	2 (10.5)	0 (0.0)	0.487
**Other Complications n (%)**						
Low debit	1 (1.8)	0 (0.0)	1.000	-	-	-
Wound infection	2 (3.6)	2 (9.1)	0.315	1 (5.3)	2 (10.5)	1.000
Tracheobronchitis	2 (3.6)	0 (0.0)	1.000	2 (10.5)	0 (0.0)	0.487
Pneumonia	6 (10.7)	3 (13.6)	0.706	2 (10.5)	2 (10.5)	1.000
UTI	1 (1.8)	1 (4.6)	0.487	0 (0.0)	1 (5.3)	1.000
Sepsis	3 (5.4)	2 (9.1)	0.617	2 (10.5)	2 (10.5)	1.000
ARF	2 (3.6)	0 (0.0)	1.000	2 (10.5)	0 (0.0)	0.487
Psychomotor agitation	0 (0.0)	1 (4.6)	0.282	0 (0.0)	1 (5.3)	1.000
Delirium	0 (0.0)	1 (4.6)	0.282	0 (0.0)	1 (5.3)	1.000
Stroke (permanent deficit)	1 (1.8)	0 (0.0)	1.000	-	-	-
AMI	1 (1.8)	0 (0.0)	1.000	-	-	-
Mesenteric ischemia	0 (0.0)	1 (4.6)	0.282	0 (0.0)	1 (5.3)	1.000
Atrial arrhythmias	9 (16.1)	2 (9.1)	0.719	4 (21.1)	1 (5.3)	0.340
**Death n (%)**						
Hospital death	2 (3.6)	1 (4.6)	1.000	1 (5.3)	1 (5.3)	1.000
30-days	2 (3.6)	1 (4.6)	1.000	1 (5.3)	1 (5.3)	1.000

AMI=acute myocardial infarction; ARF=acute renal failure; AVR=aortic valve repair; UTI=urinary tract infection.

**Table 4:** Late postoperative data for the patients in the AVR and non-AVR groups. The patients were paired according to their propensity scores.

	Original Cohort			Paired Cohort		
	Repair			Repair		
Variables	No (n=54)	Yes (n=21)	*p*	No (n=19)	Yes (n=19)	*p*
Thromboembolic complications, n (%)	1 (1.8)	0 (0.0)	1.000	-	-	-
Endocarditis, n (%)	1 (1.8)	0 (0.0)	1.000	-	-	-
Late reoperation, n (%)	1 (1.8)	0 (0.0)	1.000	-	-	-
**Time between pre and final**						
**echocardiogram (in months)**	48	13		23	14	
Median (25 - 75%)	(18-84)	(9-18)	0.002	(8-72)	(9-20)	0.275
LVEF (mean±SD)	0.61±0.08	0.59±0.11	0.434	0.58±0.11	0.58±0.12	0.910
LVEDV (mean±SD)	155±85	142±65	0.589	141±48	141±69	0.765
**Late cardiac insufficiency, n (%)**			0.129			0.250
FC I	0 (0.0)	1 (5.3)				
FC II	25 (52.1)	13 (68.4)		8 (53.3)	13 (81.3)	
FC III	21 (43.8)	4 (21.1)		6 (40.0)	2 (12.5)	
FC IV	0 (0.0)	0 (0.0)		1 (6.7)	1 (6.3)	
**Aortic valve function, n (%)**			0.208			0.536
absent AI	7 (15.6)	2 (11.1)		1 (8.3)	2 (12.5)	
minimum AI	4 (8.9)	4 (22.2)		1 (8.3)	4 (25.0)	
discrete AI	24 (53.3)	12 (66.7)		9 (75.0)	10 (62.5)	
moderate AI	6 (13.3)	0 (0.0)		0 (0.0)	0 (0.0)	
severe AI	4 (8.9)	0 (0.0)		1 (8.3)	0 (0.0)	
**Follow-up time (in months)**	50	3		28	3	
Median	(15-81)	(2-34)	< 0.001	(8-50)	(2-34)	0.073
**Death (30 days or more of follow-up)**	4 (7.1)	1 (4.6)	1.000	1 (5.3)	1 (5.3)	1.000

AVR=aortic valve repair; FC=functional class (New York Heart Association); LVEF=left ventricular ejection fraction; LVEDV=left ventricular end-diastolic volume; SD=standard deviation.
